# Public involvement in research within care homes: benefits and challenges in the APPROACH study

**DOI:** 10.1111/hex.12431

**Published:** 2015-12-01

**Authors:** Katherine Froggatt, Claire Goodman, Hazel Morbey, Sue L. Davies, Helen Masey, Angela Dickinson, Wendy Martin, Christina Victor

**Affiliations:** ^1^ Faculty of Health and MedicineLancaster UniversityLancasterUK; ^2^ CRIPACCUniversity of HertfordshireHatfieldUK; ^3^ School of Health Sciences and Social CareBrunel University LondonUxbridgeUK

**Keywords:** care homes, health and social care research, older people, patient and public involvement

## Abstract

**Background:**

Public involvement in research (PIR) can improve research design and recruitment. Less is known about how PIR enhances the experience of participation and enriches the data collection process. In a study to evaluate how UK care homes and primary health‐care services achieve integrated working to promote older people's health, PIR was integrated throughout the research processes.

**Objectives:**

This paper aims to present one way in which PIR has been integrated into the design and delivery of a multisite research study based in care homes.

**Design:**

A prospective case study design, with an embedded qualitative evaluation of PIR activity.

**Setting and participants:**

Data collection was undertaken in six care homes in three sites in England. Six PIR members participated: all had prior personal or work experience in care homes.

**Data collection:**

Qualitative data collection involved discussion groups, and site‐specific meetings to review experiences of participation, benefits and challenges, and completion of structured fieldwork notes after each care home visit.

**Results:**

PIR members supported recruitment, resident and staff interviews and participated in data interpretation. Benefits of PIR work were resident engagement that minimized distress and made best use of limited research resources. Challenges concerned communication and scheduling. Researcher support for PIR involvement was resource intensive.

**Discussion and conclusions:**

Clearly defined roles with identified training and support facilitated involvement in different aspects of the data collection process. This can also ensure that vulnerable older people who participate in research have a positive experience that reinforces the value of their views.

## Background

The involvement of members of the public and patients in research is well developed in the United Kingdom (UK) both in service development and research,^1^, ^2^, ^3^ reflecting the growth of ‘user groups’ (especially in mental health and disability fields); wider democratic movements and the rise of consumerism in health and social care.^4^ Involvement within health and social care research is described as ‘doing research with, or by, the public, rather than to, about or for the public’ (p.6).^5^ The term public, in this paper, refers to people who have experiences as patients and as family carers for patients.^6^

A key strength of public involvement in research (PIR) is proposed to be improved recruitment to studies, ensuring that research questions reflect the priorities of those studied and helping findings to be meaningfully disseminated.^7^ Wider consultations have also meant that the public have been involved in decisions about research foci and design^3^ There is a small but growing body of work that has considered the role of older people in research^8^ and more specifically in data collection and fieldwork activities.^9^, ^10^

Whilst some attention has been paid to researcher preparation for research in care homes,^11^, ^12^ PIR activity in care homes is less well developed, although examples now exist in the UK.^9^ It is recognized that residents in care homes are a group that require additional time to recruit and achieve meaningful consent. There is evidence to suggest that peer support and facilitated discussions can improve PIR engagement within a study and provide rich data.^9^ Building upon the PIR work previously undertaken by some of the study team,^9^ this paper presents one way in which PIR has been integrated into the design and delivery of a multisite research study based in care homes and considers reported benefits alongside the support required to achieve engagement. Four dimensions of user involvement are used to describe the processes adopted in the study, with respect to the context, methods, roles and outcomes.^13^

## Context: the APPROACH study

In the UK, care homes without on‐site nursing rely on primary care services for access to generalist and specialist medical and nursing services. The APPROACH (Analysis and Perspectives of integrated working in PRimary care Organisations And Care Homes) study aimed to collect and synthesize evidence about working between primary health‐care and care home providers and develop a typology of integrated working to inform future service development and research in these settings. Phase one entailed a systematic review of the literature on the effectiveness of health‐care interventions in care homes^14^ complemented by a national survey of care home managers about their experiences of integrated working.^15^ Phase two was an in‐depth case study in three sites that compared three different approaches to integrated working in six care homes. Residents in each setting were followed for a year to record any changes in their health, treatment and service use. Data collection included: serial resident interviews (*n* = 84) with 58 residents, resident notes reviews (*n* = 133), care home staff interviews (*n* = 53), primary care staff interviews (*n* = 57), one‐off relative interviews (*n* = 3) and stakeholder interviews (*n* = 12); care home and primary care staff interviews were either conducted one‐to‐one or in focus groups (*n* = 8). PIR activity was undertaken in both the management of the study and throughout the process of undertaking the study. This latter element of the study was evaluated to understand the process of PIR in care home research.

## Methods

A qualitative evaluation of PIR activity was embedded in the APPROACH study. The internal evaluation approach was designed to be participatory and formative so that PIR members could be actively involved in reviewing the PIR process throughout the study and improve mutual learning.^16^

### Setting and sample

PIR work was undertaken within project management meetings and at each of the three sites used in the APPROACH study with PIR involvement in data collection in the six care homes involved in the study. Six PIR members were involved, one in project management and five members in the three sites.

### Data collection

All PIR meetings were documented, and notes of the content of discussions recorded. A structured reflective template was completed by PIR members and researchers following each data collection visit to a care home. This provided a record of the number of PIR engagements in fieldwork recording date, time, focus of, and actions arising from, the activity.

### Analysis

The template data was typed up into a word document and analysed through the identification of descriptive codes which were then grouped into three of the dimensions of user involvement areas outlined above: methods, roles and outcomes. Key issues identified about the process of undertaking PIR activity were collated and fed back to the research team and PIR members at the final cross‐site project meeting. The main benefits and challenges were agreed by the whole project team.

Ethics approval was granted by the Essex 2 NHS Research Ethics Committee (REC reference 10/H0302/14).

## Results

### Methods of public involvement in research (PIR) work

PIR work within the APPROACH study was integrated throughout the research, in the preparatory, execution and translational phases,^17^ from project design to dissemination and in the study management and sites (Table 1). The work was supported by dedicated time to build and support PIR activity.

**Table 1:** Public involvement in different research phases

Research phase	Type of involvement
Preparatory	Older members of the Public Involvement in Research (PIR) group, at the Centre for Research in Primary and Community Care (CRIPPAC), University of Hertfordshire, with direct experience of care home engagement, were involved in the development of the funding proposal
Execution	One PIR representative participated in the study's Steering Committee overseeing its delivery to time and focus.^17^ Five PIR members assisted in fieldwork activity involved at each study site and were involved in recruitment and data collection processes
Translational	Attendance by PIR representatives at a final Validation event

A formal, transparent approach to the recruitment of PIR members was adopted in each of the three university sites undertaking case study work. Individuals were sought with prior experience of engaging with staff and older people in care homes, either through personal experience, prior employment, or involvement in previous research projects. Written information was prepared about the study and distributed to local public involvement groups. Two people per site were recruited for this work (one person withdrew from Site 3 owing to ill‐health and personal challenges with research in this setting). Prior to confirmation of involvement, governance processes such as mandatory checks for criminal records and the issuing of honorary contracts with the respective universities were followed. All PIR members were eligible to receive travel expenses and honorariums as determined by the university site practices, based on national guidelines for user involvement.^6^, ^18^

The individual role that PIR members took in the study was a negotiated one and was iteratively developed at the start of, and during, the study. The research team had ideas based on previous experiences in another study,^9^ but these were discussed with the PIR members before a decision was made about their activity in the project. The fieldwork activities that PIR members undertook were: recruitment, interview facilitation, resident support and researcher support. At all three sites PIR members assisted in the introduction of the study to care home residents, either in a group meeting, or in individual discussions with residents, or in both.

Following the initial recruitment visits to care homes, the PIR member accompanied the researcher to support resident interviews. Prior to the interview, the PIR member spent time with the residents reminding them about the research and the interviews, which facilitated the researcher's consenting process and subsequent engagement with the resident during the interview. After the interview, the PIR member revisited the resident to check they were happy with what had happened. This role was both a support to the resident and the researcher. PIR visits with the researcher increased the project presence within the care home during the study and also facilitated on two occasions (Site 2) the undertaking of a more than one resident interview per visit. It also supported governance through ensuring that residents had the opportunity to ask questions or have points raised after the interviews were completed. PIR members also supported researchers to conduct two focus group interviews with care home and primary care staff. Their role included welcoming focus group participants on arrival and assistance with distributing information sheets with attached consent forms, which were gathered and checked by the researcher. The PIR member also acted as a recorder, note taking to record process, dialogue and interactions in the group's discussions. Immediately afterwards the research and PIR member reflected on the discussion and both noted points of interest, insights and initial issues.^19^

Support and training for PIR activity was delivered in two ways: in locality meetings at each research site and in cross‐site meetings for the five PIR members across the three sites. This created a working relationship with one researcher at each site, a wider peer support group, as well as fostering relationships with the wider team. At the first meeting, held jointly with the wider project team, the study and PIR role were introduced to the PIR members. The second meeting, which involved the PIR members, PIR project site leads and researchers from each site, discussed experiences and expectations of the work and identified future areas of work for PIR representatives. The third meeting followed a period of involvement in data collection, and was an opportunity to reflect on the work undertaken to date, identify the learning and challenges encountered, and to make plans for involvement in dissemination activity.

Site‐specific processes for preparation and support of PIR members were tailored to accommodate the different previous experiences with care homes and involvement in research of the PIR members. For example, all sites held preliminary meetings with PIR members to introduce them to the project. PIR members at Site 1 were part of the user group that had reviewed the proposal, so required less preparation than at Site 3 where a session about the study was delivered to the PIR members who were newly recruited for the study. On‐going communication occurred through e‐mail, telephone and face‐to‐face meetings. In Site 1, where the PIR members were part of a larger group involved in a range of on‐going studies, informal conversations about the project occurred around other regular meetings. In Site 2, five face‐to‐face meetings were held between the two PIR members and the researcher to review activity, plan further engagement and answer questions. At Site 3, the initial training meeting was followed by one further meeting to review the work. At all sites short preparation and follow‐up support meetings prior to, and after, each fieldwork visit were provided. PIR members visited care homes with the researchers to explain the study to older residents and to support recruitment and interviews.

### Review of PIR processes

A number of benefits and challenges were identified for individuals (PIR members and researchers) and the process of undertaking the research study. At the level of project management, the PIR member who engaged with the project management through membership of the study steering group identified from his perspective specific ways in which he had influenced the study design. This concerned the format of interviews with family members and also in the development of an organizational map of the study to map its different components.^20^

Operationally, the five PIR members who were actively involved in fieldwork did this across all six care homes in the three sites. Six researchers undertook fieldwork with five PIR members on 17 occasions (Table 2).

**Table 2:** PIR fieldwork activity by site

PIR fieldwork activity	Site 1		Site 2		Site 3	Total visits (participants)
	PIR 1	PIR 2	PIR1	PIR2	PIR1	
Care home resident recruitment visits			1 visit	3 visits	1 visit	5
Care home resident interviews	2 visits 4 interviews	1 visit 2 interviews		3 visits 10 interviews	1 visit 1 interview	7 (17)
Care home staff focus group		1 focus group 4 staff	2 focus groups 9 staff			3 (13)
Primary care staff focus group	1 focus group 4 staff		1 focus group 9 staff			2 (13)
Total visits (participants)	3 (8)	2(6)	4 (18)	6 (10)	2 (1)	17 (43)

Seventeen (20%) of interviews with residents on ten occasions, and 13 (23%) of interviews with primary care staff were supported by a PIR presence. PIR members were involved in focus groups with primary care and care home staff on four occasions (twice each at Site 1 and Site 2). The extent to which PIR members were involved at each site varied, with most engagement at Site 2, and least at Site 3. The key issues identified about the process of undertaking PIR activity reflected both facilitators and challenges arising from the fieldwork activity (Table 3).

**Table 3:** Positive and challenging experiences of PIR members

What went well?	Working relationships Establishing clear roles within project Extra support Extra resource	Working together as PIRs and researchers during fieldwork in care homes Feeling part of the project To facilitate the inclusion of frail, elderly ‘vulnerable’ people as research participants More people present during data collection
What was more difficult?	Environment and communication Seeing and hearing about resident's distress Practicalities of arranging PIR involvement ‘Holding’ PIR work by researcher	Potential confusion of roles and responsibilities at time of visit Ease of hearing and talking to residents in communal areas or where residents have hearing problems Hearing or observing situations that do not look or feel right Short notice often given by care home for visits and therefore little time for PIR members to respond Multiple activities researcher has to hold when working with PIR members in terms of time and emotional support needed to provide oversight and supervision for another person alongside data collection activities

### Benefits of PIR involvement

Positive features were identified about the way the PIR work was structured in the project. The identification of clear roles and activities ensured that PIR members felt a part of the project team. In terms of impact upon data collection, PIR members were an extra resource for the project, enabling the researcher to focus on the conduct of interviews, knowing the residents were supported afterwards and conversations could be continued after the interview. This was particularly useful in the care home setting where resident interview appointments needed to be scheduled at convenient times to fit with care home routines and other activities, as it enabled residents who wanted to do so, to talk further after interviews, as this researcher described about what went well: ‘Having additional people to explain study to residents and answer questions’. (Site 1: researcher 1). This practical involvement also provided PIR members with context specific insights that informed subsequent discussions about analysis and findings between team members.

With PIR support, researchers were able to undertake a more intensive schedule of interviewing in narrow windows of opportunity, with PIR members reminding residents about the research prior to an interview, particularly important if there had been a gap in time between initial information about the study being read and the actual date of the interview, as described here ‘I also enjoyed feeling that I was of use both to the researcher and residents, some of whom had not fully heard or understood that was said’. (Site 2, PIR1). The PIR members also provided follow‐up support for residents if this was required, with less demand upon care home staff to meet this need.

### Challenges of PIR involvement

Practical challenges faced during the fieldwork by the PIR members and the researchers were how to respond when residents had difficulty communicating or were distressed. The care home environment was not always conducive to completing confidential interviews in a peaceful and undisturbed environment. These issues were discussed and when appropriate followed up with research staff in the post‐fieldwork debriefing meeting. As a consequence of undertaking site visits PIR members also reflected on their own circumstances: ‘Made me realise that I am lucky to have good health’ (Site 3: PIR 1).

The greatest challenge to PIR involvement was one of scheduling, as described by this researcher, when asked about the challenges: ‘Co‐ordinating joint visit arrangements, together with fitting in with care home’. (Site 2: Researcher 1). Arrangements for visits were often only confirmed by the care home at short notice, which often meant the PIR members already had other commitments. Distance to sites could also vary and if further away increased the time commitment for participation. Hence, PIR members were only present and able to assist at 17 of the 84 (20%) resident interviews.

Whilst the presence of PIR members during fieldwork visit was a support for the residents and the researcher, it did require that researchers paid attention to the activities undertaken by PIR members. Alongside their own work, undertaking the interviews, this added another level of complexity in an already busy environment. Finally, researcher time was also needed to plan, organize and record PIR site meetings, and to follow‐up action points or support needs emerging from these.

## Discussion

The integration of PIR work into the APPROACH study described here illustrates the four essential components of patient and public involvement in research described by Shippee *et al*.^17^: patient and service user initiation, building reciprocal relationships, co‐learning and assessment and feedback. The involvement of public representatives, with experience of research in care homes, in the development of the funding protocol ensured a PIR perspective was integrated into the research design at the start of the research. The building of reciprocal relationships was an on‐going process that developed throughout the study, varying by site. Site 3, which had least PIR activity was the site where the researchers had limited previous relationship with PIR members in the care home context, whereas at Sites 1 and 2, there were established PIR groups and relationships to build upon. In the execution of the study, the processes of post‐fieldwork debriefing meetings, on‐site and cross‐site meetings for the training and support of PIR members ensured the building of reciprocal relationships, offered opportunities for co‐learning with the researchers and between the PIR members at different sites and provided an opportunity for reassessment of the processes and feedback. The final Validation event, the translational phase of the study, was a further opportunity for evaluation and feedback of the PIR perspectives.

Undertaking research in care homes is challenging,^11^, ^21^ and researchers need to pay attention to their experience, skills and preparedness; and to their coping resources in the face of their own ageing and witnessing older people's distress in the setting.^11^, ^22^ The issues for PIR members involved in fieldwork in the care home setting are similar to those faced by researchers, with the need for appropriate preparation to understanding the culture of care in care homes, and also a consideration of how to cope with communication challenges and seeing resident distress. The issue of facing their future ageing, prompted by being in the care home environment, was raised by two PIR members including the PIR member in site 3 who withdrew from working on the study. Maybe being older themselves, with more experience in visiting care homes, meant that the dissonance between self and what was seen was less pronounced, than for younger researchers.^10^ A framework of accountability, as recommended when working with older people as researchers^23^ was provided in this study through clear role definition, negotiated responsibilities and tailored training, alongside the on‐going support.

Whilst the PIR formative evaluation documented the processes of preparation, on‐going support and perceived outcomes, the cost effectiveness of this approach has not been documented. Given that only 20% of resident interviews were supported by a PIR presence, a commitment to PIR may be on value and practical grounds rather than on the financial benefits of the involvement.^24^ The presence of a second person in the care home during fieldwork did obviate challenges experienced in other studies, for example, around recruitment.^25^ As PIR practices becomes more embedded and normalized in research in care homes then their impact on recruitment and engagement of residents may become more evident.

The volunteer nature of PIR activity combined with the need to adhere to project timelines means there is little flexibility to ensure the maximum involvement of the PIR members, as has been noted elsewhere.^8^ A larger pool of PIR members to draw upon might ensure a greater likelihood of a PIR member being available to assist in data collection visits, but would have a greater potential cost in terms of training and funding of time. A move to formally engage PIR members as research team members may address this, but may change the role they play in the study.

Training and support for PIR members has generally been driven by the needs of individual studies.^26^ More formal training is being developed for PIR work,^26^ and if implemented more consistently may address some of the challenges of providing appropriate issues of preparation and on‐going support identified in this study. However, in the two sites where PIR members were drawn from pre‐existing groups, their prior training as part of the group had been necessarily generic as PIR members could be involved with a number of diverse studies. To ensure appropriate preparation of PIR members some study specific training and support will always need to be identified and provided. Funding for these generic, or study specific, training is not always costed into funding bids, and therefore, the cost of PIR work is generally underestimated.

A possible framework for structuring the process of public involvement work in care homes research is proposed (Table 4). Using the components identified by Shippee *et al*., the operational activities that need to be undertaken to support PIR in care homes have been identified. The initiation of public involvement activity requires identification of a recruitment process for PIR members that uses a clear person specification in terms of prior experience required for the study. Where possible utilizing existing networks for recruitment reduces the time required to identify people but also is a good basis for the building or reciprocal relationships. The identification of prior relevant experience about public involvement activity, the recruitment of research participants and experience in the care home setting as either a patient, family members or worker feeds into the training needs to be met. A clear role definition for PIR members that has been collectively developed and agreed is the basis for future working relationships. Attention to safeguarding requirements is also needed.

**Table 4:** Managing PIR activity throughout a project

Public involvement initiation	Building reciprocal relationships	Co‐learning	Assessment and feedback
Identification of recruitment process for PIR members using person specification; Where possible utilize existing networks	Negotiate and agree responsibilities within project	Tailored training about: research activities care context reflection process Undertaken at start and during project, as required	Agree inbuilt internal evaluation process Regular whole PIR team and site meetings Use of a template to facilitate reflection on fieldwork activity
Identification of prior relevant experience re public involvement activity recruitment of participants experience in care setting	Establish regular meetings for site team		
Clear role definition articulated and agreed	Provide ongoing support at different levels in project: whole team site team (if required) fieldwork visits

The building of reciprocal relationships is an on‐going process, begun at the initiation of the project and requires attention to the different researcher and PIR responsibilities within project. The establishment of regular meetings for site team for communication and specific times for the provision of on‐going support at different levels in project are required. This is at all levels of the project: across the whole team, within site team (if a multicentre study) and also before and after fieldwork visits, and includes consideration of personal ageing and mortality. Co‐learning within the project team is linked to the provision of tailored training about research activities, the care context where fieldwork is to be undertaken and processes of reflection to be fulfilled. This training is undertaken at the start of, and during the project, as required. The process of assessment and feedback is best held within the development of a collectively agreed inbuilt internal evaluation process, that is integrated into the regular whole PIR team and site meetings. The use of a template to guide reflection is helpful.

## Conclusions

The involvement of PIR members within the APPROACH study occurred throughout the research process, across three sites. A number of activities and roles can be undertaken in the execution of research by members of the public to support research in care homes. With established relationships, clear role definition, appropriate training and support, and team work, the APPROACH study team were able to facilitate PIR work across three geographically dispersed sites in a way that enhanced aspects of recruitment and data collection with potentially vulnerable participants in care homes. A framework for public involvement work in care homes research is proposed.

## Funding source

This research was supported by the National Institute for Health Research Service Delivery and Organisation programme (project number 08/1809/231), Southampton, Hants, UK. The views and opinions expressed therein are those of the authors and do not necessarily reflect those of the NIHR SDO programme or the Department of Health.

## Conflict of Interests

No conflict of interests have been declared.
